# Understanding colour retention in red chilli pepper fruit using a metabolite profiling approach

**DOI:** 10.1016/j.fochms.2021.100013

**Published:** 2021-01-26

**Authors:** Harriet M. Berry, Florence Lai, Aniko Kende, Daniel V. Rickett, Charles J. Baxter, Eugenia M.A. Enfissi, Paul D. Fraser

**Affiliations:** a School of Biological Sciences, Royal Holloway, University of London, Egham, Surrey TW20 OEX, UK; b Syngenta, Jealott’s Hill International Research Centre, Bracknell, Berkshire, RG42 6EY, UK

**Keywords:** *Capsicum*, Carotenoids, Colour, Retention, Post-harvest storage, Volatiles, Metabolomics, 2,6 –Nonadienal (PubChem CID636687), 2-Hexenal (PubChem CID5281168), 4-Oxononanal (PubChem CID156288), *trans*-2-Nonenal (PubChem CID5283335), 6-Methyl-5-hepten-2-one (PubChem CID9862), β-Ionone (PubChem CID638014), Geranyl acetone (PubChem CID1549778), Dihydroactinidiolide (PubChem CID6432173), Linoleic acid (PubChem CID5280450), Linolenic acid (PubChem CID5280934)

## Abstract

Carotenoids are the pigments responsible for conferring the characteristic deep red colour to chilli pepper. The post-harvest retention of this colour is a key trait that governs the price of the produce. Determining colour retention and the associated underlying biochemical mechanisms are important issues that require investigation. In this present study, the ability of image analysis to determine colour change in ground chilli fruit was evaluated. This method enabled differentiation of extreme retention phenotypes whilst also reducing the duration of storage required to make accurate determinations. The analysis of volatiles indicated different levels of lipid and carotenoid derived volatiles in lines with different retention properties. Metabolite profiling of intermediary metabolism supported these findings, with increased levels of unsaturated fatty acids present in lines with low retention properties. Collectively, these data have led us to propose that in chilli fruit lipid peroxidation is one of the progenitors of carotenoid degradation.

## Highlights

• Image analysis can be used to speed up identification of high retention phenotypes.
• High and low retention phenotypes can be identified by their volatomes.
• Low retention lines have increased unsaturated fatty acids.
• Post-harvest colour loss in chilli fruit involves lipid peroxidation.

## 1 Introduction

Food colour is an important consumer trait, with the consumer associating the richness of colour with fruit quality and nutritional value. More recently there has been a preference for the use of natural sustainably sourced colorants in food, eliminating the use of artificial/ chemically synthesised colorants (Arimboor et al., 2015). Carotenoids are natural pigments that provide the red, orange and yellow hues to fruits and vegetables. In addition to their ability to confer colour, they are potent bioactives. There is now a wealth of scientific evidence supporting the consumption of carotenoid rich fruits and vegetables as being beneficial to human health (Fraser, 2004, Giuliano, 2017).

Apocarotenoids are organic compounds which are derived from the non-enzymatic and enzymatic oxidative cleavage of carotenoids. The enzymes responsible for the formation of apocarotenoids belong to a family of carotenoid cleavage oxygenases (CCOs), which include carotenoid cleavage dioxygenases (CCDs) and 9-*cis*-epoxycarotenoid dioxygenases (NCEDs) (Sun & Li, 2020). The pepper genome contains 11 CCO genes which are orthologous to tomato, with the *CaCCD4a* specific to fruit ripening (X. H. Zhang et al., 2016). In addition to this, lipooxygenase (LOX) enzymes involved in the oxidation of lipids to create C5 and C6 volatiles play a role in apocarotenoid production whereby the free radicals produced are quenched by carotenoids leading to their cleavage (Gao et al., 2019). Apocarotenoids can be either volatile or non-volatile in nature and are believed to mediate physiological processes in plants and humans (Rivers et al., 2019). In addition to their status as cellular modulators, apocarotenoids contribute to the aroma and taste of foods (El Hadi et al., 2013). On the contrary, increased apocarotenoid production can have detrimental effects on colour intensity. Thus, when breeding new crop varieties a balance is required to ensure colour and nutritional quality is achieved without compromising the contribution of apocarotenoids to taste and aroma. These parameters are particularly important in fruits and vegetables where colour intensity and differentiation are key determinants of consumer quality.

Chilli fruit is a good example where intensity and retention of colour are key commercial parameters dictating the premium of the produce. The intense red coloration of ripe chilli fruit is due to the presence of carotenoid pigments, predominantly the presence of capsanthin, a carotenoid that is virtually unique to *Capsicum* fruits (Berry et al., 2019). Red chilli peppers are the oldest, most popular and economically important natural food colourant known, having an average annual production of 2.5 million tonnes (Arimboor et al., 2015). They can be harvested at mature green or red ripe stage, eaten fresh or they can dried, ground, stored and used as a spice or food colorant. Red chilli peppers are grown predominantly in India, South East Asia and the Americas for the production of chilli powder, or paprika. In India, after ripening and production of red ripe fruit, the chilli peppers are harvested and air dried under natural sunlight. Once the chillies are dry they are stored at 10 °C in the dark until they are sold. Some varieties of chilli are susceptible to colour loss during storage. Therefore, the ability of the chilli pepper fruit to retain a desirable deep red colour throughout storage is a key determinant for optimal price.

Although there has been intense efforts to develop colour intensity in red chilli fruit (Berry et al., 2019, Cervantes-Paz et al., 2014) far fewer publications are dedicated to colour retention during post-harvest scenarios. This is surprising considering one of the long-term goals of the food industry is the development of chilli varieties with improved colour retention properties. The present study has contributed to this aim by: (i) developing and evaluating a procedure for improved logistical monitoring of colour retention in chilli diversity, (ii) determining a potential association between colour retention and carotenoid catabolism and (iii) advancing our metabolite resources in chilli to assess potential metabolome variation and its association with colour retention.

## 2 Materials and methods

### 2.1 Plant material

The chilli pepper lines were part of a colour discovery panel supplied by Syngenta and have been designated names R1-12 (Table S1).

### 2.2 Image analysis

#### 2.2.1 Camera set up

The image analysis method was based on the use of a computer vision system (CVS) to obtain the L*, a* and b* coordinates, using the red, green and blue (RGB) colour model values of a digital image, as a quantitative measurement of colour. The CIELAB colour space, defined by the International Commission on Illumination (CIE), was used to obtain the L*, a* and b* coordinates. The image analysis system was designed by Syngenta. Algorithms corrected for the image variables due to source distance, camera’s own characteristics, fruit glossiness and fruit curvature. The CVS consists of a suspended digital camera (canon EOS Digital Rebel XT with lens: Canon 10–12 mm Zoom Macro and Canon 50 mm Macro) above an imaging igloo which contained the sample to be photographed (camera 50 cm above sample). Two fluorescent lamps (Westcott SpiderLite TD5 Location kit with Westcott 27 W/ 100 V DayIt FLOU lamps) focused on the igloo to illuminate the sample. The angle from the axis of the lens and the sources of illumination were 45⁰. The light stands were 1.8 m in height and were positioned 80 cm from the imaging platform on each side. The camera was connected to the computer, where it was operated remotely. Image analysis relies on keeping all the variables constant; therefore, in order to keep the light the same intensity, the light was warmed for 20 min prior to use and the set up was behind a blackout curtain.

The software used for camera-to-computer communication was EOS Utility and the software used for image viewing and editing was Digital Photo Professional version 3.8.1. The camera was set to autofocus on the lens and manual on the dial; the lens was set to 50 mm. The remaining settings were input using EOS Utility – the settings were: Mode, manual; shutter speed, 1/6; Aperture, F14 (whole chillies) and F20 (powder); ISO, 100; white balance, custom; Metering mode, evaluating; lens focus mode, autofocus; image quality, large fine; image type, .JPG.

#### 2.2.2 Image calibration

The camera was calibrated to a white balance (Photoflex EZY grey balance) and colour card (X-Rite Digital Colour Checker). The white balance measured the RGB saturation at the edge and middle of the image to ensure uniformity. A black background was used to image powdered chilli pepper and a white background was used to image whole chilli peppers due to the shiny surface of the chillies reflecting the black background.

#### 2.2.3 Sample preparation

The 12 chilli pepper lines from the discovery panel were grown in mesh net houses in Aurangabad, Syngenta India Ltd. They were harvested at ripe red stage and after sun-drying (3 weeks) were sent as dry, whole fruit (20 per line). The fruit was ground using a blender and the chilli powder was incubated on an assay plate (12 wells) for 6 weeks under different light and temperature conditions. The conditions were 15, 20, 30 and 40⁰C in 12/12 hr light/ dark cycle (70–80 µmol. m^−2^. *sec*^-1^). 30⁰C under UV (constant) and 30⁰C under blacklight (constant).

#### 2.2.4 Analysis

Images were taken at the start of the experiment and every week, for 6 weeks. A macro was used to convert the RGB values of the image into L*, a* and b* coordinates. The macro was run using ImageJ and script written and supplied by Rob Lind, Syngenta (Schneider et al., 2012). A reading was taken for each well on the plate (n = 12). Each See equation below for the calculation of total colour change.

$$\Delta E=\sqrt{{\left(L_{F}-L\right)}^{2}+{\left(A_{F}-A\right)}^{2}+{\left(B_{F}-B\right)}^{2}}$$

### 2.3 Volatile analysis

#### 2.3.1 Sample preparation

The discovery panel was grown under temperature and light controlled conditions from seed in the greenhouse at Royal Holloway University, UK (25 °C day/ 15 °C night; 110 µmol. m^−2^. *sec*^-1^; 16/8 h light/ dark cycle). Lines R1, R2, R3, R4, R7, R8, and R9 were selected due to their colour retention phenotypes, performance throughout the image analysis experiment and overall vigour in the greenhouse.

Fresh material: Chilli pepper juice extract was produced by adding water (150 mL) to fresh fruit (50 g) and homogenising in a blender (4 min). Aliquots (1.5 mL) were added to Cryo. s freezing tubes (4 mL; Greiner Bio-One, UK) and frozen in liquid nitrogen. Six technical replicates (created from pools prepared from 4 plants) from each independent chilli line were frozen. The ratio of dry weight to water was calculated by weighing material before and after freeze-drying.

Dry Material: Chilli peppers (50 g) were dried for three weeks in an incubator (35 °C). The same ratio (as fresh material) of water was then added to them and they were homogenised in a blender (4 min). Aliquots were made and samples were immediately frozen in liquid nitrogen.

Volatile analysis was carried out using silicone rod extraction thermal desorption – gas chromatography – mass spectrometry (TD-GC–MS) by a method described in detail by Kende et al., (2019). Semi-quantification was achieved by comparing the peak areas of the target compound to a response curve corresponding to the internal standard 1,4-dichlorobenzene (20 μL; 18.75 μg/mL in methanol). Linearity was checked and maintained using the extraction parameters described. Compounds which did not appear in all replicates of the chilli line being analysed were removed and the amounts calculated (ng/ μL) were log transformed in preparation for principle component analysis.

### 2.4 Metabolite analysis

#### 2.4.1 Sample preparation

Lines R1, R3 and R7 were selected for metabolite analysis, 3 fruits (pooled) from 4 plants were analysed for each line (n = 4). Ripe chilli peppers were harvested, deseeded, frozen in liquid nitrogen and freeze dried. The dried material was then homogenised to a fine powder.

Methanol (400 µL) and distilled water (400 µL) were added to ground chilli powder (10 mg) and vortexed. The samples were then rotated at room temperature (1 hr). Chloroform was added (800 µL) and the samples were vortexed and centrifuged at 12000 rpm (5 min). Both phases, polar and non-polar, were collected. The polar extract (20 µL) was transferred to a glass vial (Agilent) and ribitol standard (10 µL; 1 mg/mL in methanol) was added. The methanol was evaporated using a rotary evaporator (GeneVac Ez-2 plus; 1–2 hr) on HPLC fraction setting. Myristic-d_27_ acid (1 mg/mL in chloroform; 10 µL) was added to the non-polar phase and the rotary evaporator was used to evaporate the chloroform on low bp setting (40 min). Samples were stored at −20 °C until derivatisation: Methoxyamine-HCl (MEOX; 30 µL; 20 mg/mL in pyridine anhydrous) was added to the samples and incubated at 40 °C (1 hr), then N-methyl trimethylsilyl trifluoroacetamide (MSTFA; 70 µL) was added and incubated at 40 °C (2 hr).

Gas chromatography-mass spectrometry (GC–MS) was carried out using an Agilent 7890B gas chromatograph system with a 5977A MSD. Samples were injected (1 µL) with a split/ splitless injector at 290 °C with a 20:1 split as previously described (Enfissi et al., 2010). The internal standard was used to retention time lock the samples. The GC oven was kept at 70 °C (4 min) before ramping at 5 °C/min to 310 °C. This final temperature was maintained (10 min) making a total of 60 min. The GC–MS interface was set (290 °C) and the MS ran in full scan mode using 70 eV EI + and scanned from 50 to 800 D. To identify the compounds in the chilli pepper profile an MS library was constructed from in-house standards, in addition to the NIST 11 MS library. To facilitate the determination of retention indices (RIs) a retention time calibration on all standards was carried out. Thus RIs and MS allowed identification by comparison with the MS library. AMDIS V2.71 was used to identify peaks and create a report. The reports generated by AMDIS were combined with ID align and transferred to Excel to normalise the data using the internal standards (Table S2). Compounds which did not appear in all replicates of the chilli line analysed were removed in preparation for principle component analysis.

### 2.5 Statistical analysis and data visualisation

#### 2.5.1 Image analysis

One-way ANOVA with Tukey post-hoc test was carried out using XLSTAT 2020 (Addinsoft, Paris, France) with *P* ≤ 0.05. Significance was grouped across the panel.

#### 2.5.2 Volatile and metabolite analysis

The log transformed, semi-quantified amounts (TD-GC–MS) and normalised peak areas (GC–MS) were analysed using principle component analysis (PCA) in SIMCA P V15 (Umetrics AB), the Q2 values were calculated automatically in this software. Values of 0.3–0.9 were obtained indicating a valid predictable model. Statistical differences (*P* ≤ 0.05) between individual components were identified using a pairwise comparison with Student’s *t*-tests. These values were determined for the metabolites and volatiles and plotted in a heat map using excel (Microsoft office 2013). These data were then overlaid on a pathway diagram using BioSynLab (Royal Holloway, University of London) as a template and then edited to integrate data from GC–MS and TD-GC-M.

## 3 Results and discussion

### 3.1 Development and evaluation of a rapid imaging procedure for colour retention assessment.

Individual perception in conjunction with environmental factors such as light intensity and physiological parameters (e.g. fruit size, texture and shape) make visual colour classification highly subjective. Recent years have seen a trend in using CVS and image analysis to measure a variety of desirable traits in fruits and vegetables (Zhang et al., 2014). This study has explored image analysis to create a more robust procedure that could eradicate the variation that results from an individual’s perception.

**Fig. 1:**
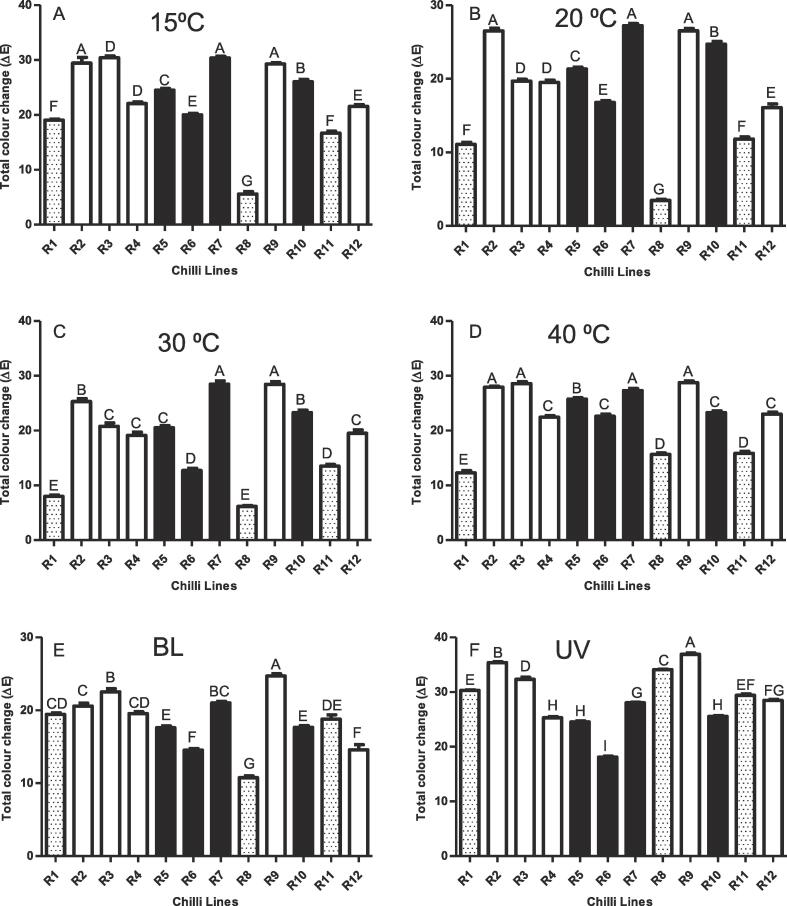
**Total colour change in chilli powder over time.** The total change in colour (ΔE) was calculated for chilli powder when incubated at different temperature and light conditions. (A) 15 °C, (B) 20 °C, (C) 30 °C, (D) 40 °C, (E) Blacklight (constant) at 30 °C and (F) UV (constant) at 30 °C. All of the temperature conditions were exposed to 12/12 hr light dark cycle for 6 weeks. Statistical analysis was performed using one-way ANOVA (*P ≤* 0.05) with Tukey post-hoc test and lines were grouped based on their significance. Errors bas represent ± SE (n = 12). Key: spotted bar, high retention; white bar, low retention; black bar, medium retention; BL, blacklight.

Industrially the determination of colour retention in chilli fruit during post-harvest storage is a long and laborious process. Presently, using whole fruit, the procedure takes up to eight months. To speed the process up the present study used ground fruit incubated at different temperatures and light regimes for 6 weeks, coupled to image analysis. The fruit incubated were from a discovery panel, supplied by Syngenta, which varied in colour retention phenotypes (Table S1). The image analysis quantified colour by recording the RGB values from an image under controlled conditions. From the CIELAB colour space, coordinates L*, a*and b* were recorded and the total colour change over time calculated (Fig. 1).

Using the image analysis approach it was found that when incubating dry, ground fruit material under different temperature (15 to 40 °C) regimes the R8 line, followed by the other high retention lines, R1 and R11, showed the lowest colour loss, with R1 becoming better at retaining its red colouration at higher temperatures when compared to the lower temperatures (Fig. 1 A-E). R8 displayed the lowest colour loss under blacklight, followed by R6 and R12. R6 underwent the lowest colour loss when compared to the other lines under UV, as well as performing well comparatively at 30⁰C. A high colour change (low colour retention phenotype) was observed with lines R2, R3, R7 and R9. These lines were consistent in their comparative loss of colour, when incubated at different temperature conditions and under blacklight (Fig. 1 A-E). However, R7 displayed less colour change under UV, while R8 had a high colour change under UV conditions (Fig. 1 F). R2 and R9 experienced the highest colour loss comparatively under UV conditions.

The approach described using image analysis to predict colour changes over storage time was able to classify extreme colour retention phenotypes. While a similar approach has been used in tomato (Lino et al., 2008), this method has not been applied to post-harvest colour in chilli pepper. The mid-range phenotypes were harder to distinguish, and in some cases perhaps need reclassification, highlighting the errors that can arise when defining colour retention visually. For example, R7 has been classified with a medium retention phenotype but in most conditions displays colour loss more similar to those with low retention phenotypes. On the contrary, R4 and R10 have been classified as low retention phenotypes but in most conditions display less colour loss than other low lines. Although the use of ground material eliminates the effect of an intact cuticle, this technique would be useful to efficiently screen large populations with the additional benefit of reducing the storage period from eight months to just six weeks. However, it is clear that more refined molecular or biochemical approaches are required to provide more in depth selection and underlying of the properties displayed by specific elite/prototype lines.

### 3.2 Is the degradation of carotenoids associated with colour retention?

In order to provide an insight into the metabolic and/or catabolic processes leading to post-harvest colour degradation in chilli, comparative volatile analysis was performed on fresh and dried chilli fruit material. Volatile (and semi-volatile compounds) were determined in fresh and dry chilli fruit tissue. The designation of fresh and dry refers to homogenised fresh ripe fruit and homogenised dry fruit (oven dried for 3 weeks), respectively. In total there were 28 volatile related compounds identified in fresh and dry fruit collectively. The chemical components from the fresh and dry fruit analysed differed in composition (Fig. S1). The dry fruit experienced an overall decrease in the percentage of aldehydes (2,6 –nonadienal, 2-hexenal, 4-oxononanal, hexanal, *trans*-2-nonenal and *trans*-2-octenal) and esters (2-methylpentyl hexanoate, hexyl 2-methylbutyrate, hexyl 3-methylbutyrate, methyl hexanoate and methyl salicylate) and an increase in ketones (6-methyl-5-hepten-2-one, β-ionone, β-ionone epoxide and geranyl acetone) and monoterpenes (dihydroactinidiolide, linalool and ocimene). The percentages of alcohols, alkanes, alkenes, pyrazines, and sesquiterpenes were very similar in fresh and dry fruit. The chilli lines used in this study showed a predominance of volatile aldehydes (approx. 30% and 20% in fresh and dry fruit, respectively) and ketones (approx. 20% and 30% in fresh and dry fruit, respectively); however, these compositions can vary considerably depending on the variety of chilli pepper analysed.

In fresh chilli pepper tissues, the most abundant compounds in ripe fruit were a mixture of fatty acid and carotenoid derived degradation products such as 2- hexenal, hexanal, *trans*-2-nonenal, *trans*-2-octenal, β-ionone epoxide and dihydroactinidiolide. The amino acid and organic aroma compounds, methyl salicylate and guaiacol, were also present. The majority of compounds identified in this study have been detected previously in chilli pepper (Bogusz Junior et al., 2012, Kollmannsberger et al., 2011, Mazida et al., 2005, Pino et al., 2006, Pino et al., 2007). However, when the dry fruit were analysed the most abundant compounds were carotenoid degradation products, predominantly β-ionone epoxide and dihydroactinidiolide as the most prevalent compounds present (Table 1; abundant compounds in bold).

**Table 1:** Volatile compounds found in fresh and dried chilli pepper fruit.

**Fresh (ng/ mL)**								**Dry (ng/mL)**						
Compound	R1	R2	R3	R4	R7	R8	R9	R1	R2	R3	R4	R7	R8	R9
2,6-Nonadienal	13.49 ± 1.13	17.21 ± 2.60	23.78 ± 18.11	5.15 ± 0.92	9.19 ± 2.33	35.10 ± 5.27	5.59 ± 1.76	6.58 ± 1.74	7.21 ± 1.79	1.44 ± 0.54	–	3.23 ± 1.28	3.72 ± 0.19	2.80 ± 1.23
2-Hexenal	**56.37 ± 12.42**	**137.51 ± 20.07**	**148.60 ± 70.33**	**73.94 ± 9.69**	**160.48 ± 34**	**110.45 ± 8.84**	**43.89 ± 6.98**	19.77 ± 5.15	13.28 ± 3.24	6.52 ± 3.33	6.27 ± 0.92	5.9 ± 1.42	70.14 ± 5.10	5.92 ± 1.38
2-Methyl- pentadecane	121.82 ± 43.33	–	16.39 ± 8.05	–	–	409.59 ± 44.03	–	34.62 ± 18.97	–	–	–	–	174.27 ± 12.76	–
2-Methylpentyl hexanoate	147.06 ± 97.06	43.55 ± 15.79	11.31 ± 3.72	10.44 ± 3.34	10.24 ± 5.70	258.9 ± 44.24	17.15 ± 6.59	15.03 ± 3.54	5.46 ± 3	–	–	5.86 ± 1.47	21.5 ± 2.91	–
2-Methyl-tetradecane	172.11 ± 87.39	–	9.81 ± 4.48	–	–	366.01 ± 72.09	–	36.4 ± 19.6	–	–	–	3.74 ± 1.71	111.17 ± 41.15	–
2-*sec*-Butylcyclo- hexanone	83.21 ± 22.21	66.12 ± 12.95	37.33 ± 16.3	21.54 ± 5.87	26.31 ± 10.54	328.44 ± 54.86	39.75 ± 7.04	44.36 ± 24.11	5.44 ± 2.35	–	–	–	49.24 ± 16.01	3.06 ± 1.41
3-Isobutyl-2-methoxypyrazine	2.28 ± 0.4	6.49 ± 0.42	13.23 ± 3.07	1.64 ± 0.05	10.23 ± 0.73	2.03 ± 0.11	3.45 ± 0.24	1.06 ± 0.29	2.72 ± 2.72	2.87 ± 0.41	0.9 ± 0.11	3.08 ± 0.3	0.9 ± 0.05	1.79 ± 0.23
4-Oxononanal	79.70 ± 46.75	26.53 ± 14.61	30.83 ± 10.52	10.48 ± 4.35	19.11 ± 8.64	223.09 ± 60.01	16.33 ± 9.56	22.65 ± 12.73	–	–	–	–	40.20 ± 13.63	–
6-Methyl-5-hepten-2-one	28.39 ± 2.21	47.22 ± 3.97	27.46 ± 1.34	25.02 ± 1.11	24.94 ± 1.68	21.8 ± 0.77	20.24 ± 1.36	39.51 ± 1.94	135.85 ± 25.52	35.11 ± 3.16	29.29 ± 0.45	79.18 ± 9.11	56.18 ± 1.65	41.19 ± 3.82
β-Ionone	12.93 ± 5.16	11.85 ± 1.65	49.46 ± 32.7	1.74 ± 0.15	6.19 ± 0.59	11.75 ± 1.58	3.55 ± 0.33	10.61 ± 0.67	89.8 ± 9.78	9.05 ± 1.66	5.66 ± 0.25	42.4 ± 5.85	9.81 ± 0.61	13.3 ± 1.29
β-Ionone epoxide	**119.33 ± 22.1**	**319.38 ± 31.75**	**57.13 ± 8.68**	**44.33 ± 2.87**	**123.41 ± 20.19**	**23.76 ± 2.02**	**73.94 ± 14.84**	**375.3 ± 25.88**	**2579.14 ± 224.74**	**264.3 ± 30.3**	**193.05 ± 10.41**	**1253.44 ± 161.5**	**212.29 ± 10.09**	**485.37 ± 71.98**
Dihydroactinidiolide	**60.60 ± 10.57**	**206.64 ± 18.29**	**57.42 ± 9.03**	**40.61 ± 5.49**	**145.31 ± 20.13**	**25.31 ± 1.59**	**58.18 ± 3.62**	**475.65 ± 27.54**	**2454.75 ± 209.06**	**416.87 ± 48.54**	**319.39 ± 17.32**	**1461.64 ± 170.82**	**293.53 ± 10.65**	**544.96 ± 37.58**
Geranyl acetone	4.39 ± 0.93	13.73 ± 2.33	8.72 ± 6.06	4.04 ± 0.21	4.10 ± 0.48	2.22 ± 0.12	2.61 ± 0.51	6.08 ± 0.73	71.75 ± 18.28	4.2 ± 0.86	10.26 ± 0.38	31.84 ± 5.88	12.99 ± 0.31	10.26 ± 1.2
Guaiacol	**356.96 ± 113.81**	**129.79 ± 23.41**	**242.05 ± 57.04**	**237.87 ± 102.71**	**195.6 ± 54.36**	**240.49 ± 22.5**	**182.94 ± 50.67**	159.58 ± 55.92	198.1 ± 63.52	135.52 ± 21.28	157.42 ± 63.95	89.72 ± 33.87	282.39 ± 90.25	84.84 ± 18.51
Hexanal	**154.51 ± 37.62**	**184.52 ± 28.05**	**214.97 ± 97.72**	**73.35 ± 4.79**	**115.09 ± 19.43**	**400.83 ± 29.33**	**125.96 ± 19.45**	87.96 ± 26.89	34.77 ± 10.06	43.75 ± 17.22	12.11 ± 1.69	10.34 ± 2.99	239.09 ± 19.85	35.64 ± 2.65
Hexyl 2-methylbutyrate	126.55 ± 74.66	–	24.24 ± 5.66	–	–	210.43 ± 43.24	4.67 ± 0.49	4.63 ± 0.98	5.39 ± 3.41	2.46 ± 1.17	–	3.23 ± 0.38	16.78 ± 6.06	–
Hexyl 3-methylbutyrate	132.6 ± 75.81	–	26.6 ± 5.53	–	–	242.08 ± 54.45	4.29 ± 2.23	16.66 ± 9.72	5.57 ± 2.98	–	–	–	83.74 ± 34.93	–
Isocaryophillene	16.58 ± 5.76	–	191.75 ± 120.19	–	–	42.03 ± 4.31	–	5.43 ± 1.94	–	–	–	–	54.23 ± 15.42	–
Isohexanol	91.76 ± 30.7	–	895.06 ± 240.02	–	–	227.73 ± 29.96	–	119.2 ± 38.59	–	435.05 ± 164.47	–	–	340.88 ± 31.44	–
Linalool	–	46.42 ± 10.94	54.95 ± 8.4	17.64 ± 1.25	49.17 ± 7.41	–	6.59 ± 2.24	5.98 ± 1.34	90.04 ± 15	30.33 ± 6	13.56 ± 2.32	63.45 ± 8.32	3.31 ± 1.11	16.8 ± 5.49
Methyl hexanoate	0.51 ± 0.1	0.44 ± 0.08	8.35 ± 6.98	0.84 ± 0.11	0.56 ± 0.04	1.40 ± 0.85	0.58 ± 0.08	0.45 ± 0.08	0.23 ± 0.04	0.46 ± 0.06	0.32 ± 0.08	0.12 ± 0.01	0.58 ± 0.12	0.44 ± 0.11
Methyl salicylate	**96.08 ± 29.11**	**123.69 ± 18.76**	**65.46 ± 13.04**	**36.83 ± 3.66**	**89.31 ± 17.31**	**357.93 ± 16.03**	**61.09 ± 12.18**	54.62 ± 6.07	34.79 ± 6.48	46.53 ± 11.88	8.47 ± 1.13	20.11 ± 2.49	225.71 ± 17.10	50.19 ± 10.65
Ocimene	0.44 ± 0.11	1.09 ± 0.2	33.31 ± 18.63	161.84 ± 48.71	–	2.97 ± 0.41	–	0.33 ± 0.04	0.15 ± 0.07	1.85 ± 0.47	13.02 ± 1.99	–	3.58 ± 0.57	–
Trans-2-Nonenal	**295.92 ± 92.68**	**323.54 ± 89.81**	**140.33 ± 47.1**	**97.53 ± 35.12**	**169.88 ± 67.24**	**583.44 ± 178.07**	**159.55 ± 49.27**	108.75 ± 31.03	85.4 ± 16.13	43.71 ± 14.83	25.08 ± 6.4	33.32 ± 7.29	75.32 ± 14.51	67.97 ± 17.9
Trans-2-Octenal	**152.57 ± 33.7**	**195.66 ± 28.17**	**73.1 ± 13.65**	**56.12 ± 10.88**	**99.63 ± 27.39**	**217.38 ± 39.58**	**87.24 ± 14.56**	63.35 ± 12.74	59.33 ± 13.23	55.48 ± 9.36	11.37 ± 5.09	33.91 ± 8.52	56.23 ± 8.9	35.27 ± 9.93
Trans-Nerolidol	23.3 ± 9.63	10.03 ± 2.73	162.89 ± 76.66	4.07 ± 0.57	1.85 ± 0.41	38.4 ± 4.12	4.86 ± 1.47	11.39 ± 3.44	3.88 ± 1.06	22.56 ± 10.78	1.12 ± 0.11	1.64 ± 0.17	47.75 ± 5.97	2 ± 0.55
Undecatetraene	–	–	–	–	–	126.49 ± 11.27	–	–	–	–	–	–	193.83 ± 22.38	–

The most abundant compounds identified in fresh and dry fruit are emphasised in bold. Mean ± SE (n = 6). Key: -, not detected.

**Fig. 2:**
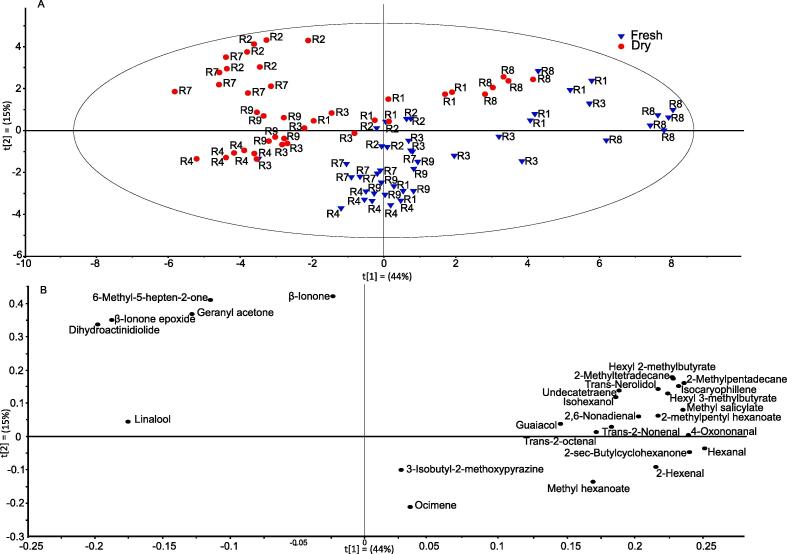
PCA of volatile compounds detected in fresh and dry chilli pepper. Volatile analysis was carried out on fresh and dry chilli peppers which had differing colour retention phenotypes (A) PCA scores and (B) PCA loadings. The values in the PCA were log transformed. The predictability of the model gave a Q2 value of 0.6 validating the approach. Key: (A) fresh fruit, blue triangle; dry fruit, red circle. (For interpretation of the references to colour in this figure legend, the reader is referred to the web version of this article.)

The fresh and dry chilli fruit volatile data were compared using principle component analysis (PCA). There was a distinct clustering between the fresh and the dry samples, on the PCA score plot (Fig. 2A). The associated loadings indicated that the lipid degradation products were driving the separation of fresh material, specifically 2,6-nonadienal, 2-hexenal, hexanal, *trans*-2-octenal and *trans*-2-nonenal (Fig. 2B); while the carotenoid and terpenoid degradation products (geranyl acetone, β-ionone epoxide, 6-methyl-5-hepten-2-one, dihydroactinidiolide and linalool) were responsible for the separation of dry fruit (Fig. 2B). The dry, high retention lines, R1 and R8, cluster to the right panel of the PCA plot closer to the fresh samples than to the dry, low retention lines. The changes in volatile compounds from fresh to dry fruit are provided in a heat map (Fig. S2). The heat map displays the transition from fresh to dry fruit and reflects the differentiating components observed from the PCA (Fig. 2). In summary, there was an increase in the amount of carotenoid derived volatiles and a simultaneous decrease in lipid derived volatiles. For example, 2,6-nonadienal and 2-hexenal were decreased in the majority of the lines analysed, with the exception of R9 (Fig. S2).

The apocarotenoids detected in the dry fruit could have been formed via the oxidative cleavage of the carotenoids by various CCDs (Simkin et al., 2004). Geranyl acetone is thought to be derived from phytoene, β-ionone and β-ionone epoxide from β-carotene, and 6-methyl-5-hepten-2-one from lycopene (Goff & Klee, 2006). Non-enzymatic free radical mediated cleavage of carotenoids generating carotenoid volatiles may also occur (Simkin et al., 2004). Although the action of CCDs could be responsible for the colour change occurring during storage in chilli fruit, more recent studies have eluded that non-CCD mediated carotenoid degradation has a greater impact (Schaub et al., 2017).

The unsaturated aldehydes driving the fresh fruit separation were derived from the unsaturated fatty acids, linoleic acid (C18:2) and linolenic acid (C18:3) (El Hadi et al., 2013). In intact fruit the lipid degrading enzymes are in separate compartments to their substrates; however, the formation of lipid derived volatiles are initiated when the cellular integrity within the chilli pepper fruit is disrupted. These events result in lipid degrading enzymes, such as lipoxygenases, coming into contact and acting on membrane and storage lipids (Galliard et al., 1977). Linoleic and linolenic acid are particularly prone to oxidation due to the presence of double bonds. The volatile aldehydes (2,6-nonadienal, 2-hexenal, hexanal, *trans*-2-octenal and *trans*-2-nonenal) formed from these unsaturated fatty acids and subsequent lipid peroxidation lead to the formation C_6_ aldehydes as identified in this study. The fatty acid peroxides produced in these catabolic processes are highly reactive and can destroy cellular membranes, proteins and macromolecules through oxidation (Stahl & Sies, 2003). In order to dissipate the actions of these reactive species, carotenoids can act as potent antioxidants. However, carotenoids are broken down in the process typically resulting in decolourisation. This has been seen in the endosperm of golden rice whereby the rice loses its ‘gold’ colour during storage due to the oxidation of β-carotene; however, down regulation of a LOX gene decreases the co-oxidation of β-carotene (Gayen et al., 2015). This has been an issue in many other biofortified crops during post-harvest storage and studies in sorghum have used tocopherols (vitamin E) to resist this degradation (Che et al., 2016). The tocopherol trend seen in ripe fruit was not correlated to colour retention phenotype. γ-Tocopherol and α-tocopherol were higher in R3 and R7, respectively, but R1 was higher in δ-tocopherol when compared to R3 (Table S2). Perhaps breeding lines with decreased LOX and increased tocopherols could add to the protection of the carotenoids and therefore boost colour retention phenotype.

The dry, high retention lines R1 and R8 appear to cluster closer to the fresh samples in comparison to the dry, low retention lines on the PCA (Fig. 2A). This suggested that these lines were showing more similar volatile compositions to the fresh samples due to the presence of higher levels of lipid derived volatiles. This was reflected when analysing the heat map showing changes in compounds from fresh to dry. In comparison to all other lines analysed these two high retention lines showed less dramatic decreases in lipid derived volatiles, particularly linolenic acid derived 2,6-nonadienal and 2-hexenal. Interestingly, linolenic acid was found to be the most abundant in the chloroplast membrane (Matsui et al., 1997); thus suggesting these high retention lines were experiencing a slower rate of degradation of chromoplast membranes. This therefore results in less carotenoid degradation as the integrity of the chloroplast or chromoplast was maintained.

**Fig. 3:**
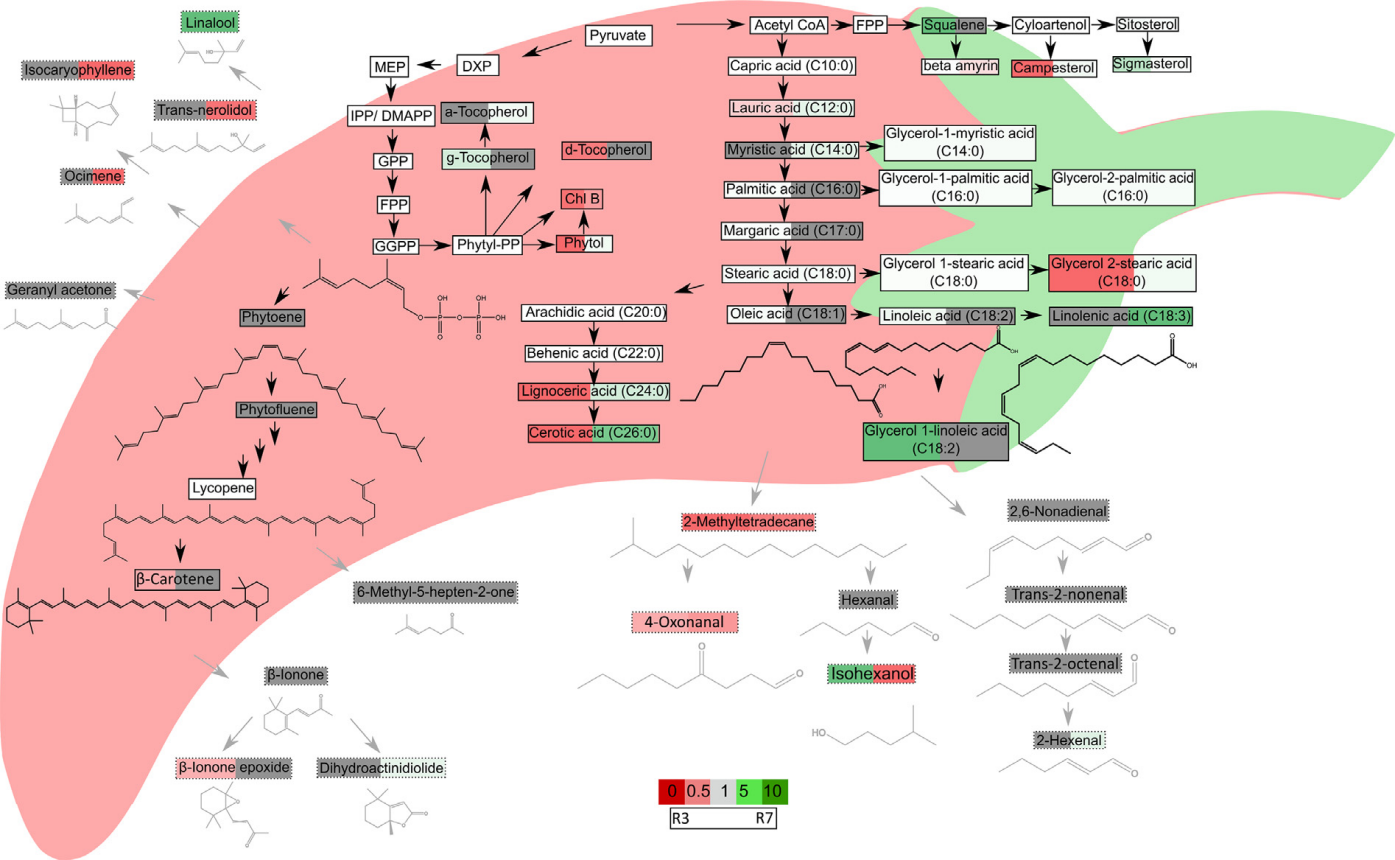
**Pathway display of metabolite changes in ripe fruit between selected lines.** Metabolite, carotenoid and volatile analyses were carried out on selected lines in fresh ripe fruit stage. R1, R7 and R3 had colour retention phenotypes of high, medium and low, respectively. R1 was used as a control and fold changes were calculated with respect to R1. Changes in metabolites were visualised over pathways for a global look at differences in metabolism. Changes are indicated by: white, not present; grey, not significant; green, significant increase; and red, significant decrease. Significance based on *P ≤* 0.05 (n = 4). R3 represents the change with respect to R1 on the left hand side of the box and R7 represents the change with respect to R1 on the right hand side of the box. Metabolite boxes with dash outline and grey structures signify volatile compounds and full line and black structures signify metabolites. (For interpretation of the references to colour in this figure legend, the reader is referred to the web version of this article.)

The carotenoid content in these lines has been studied in detail previously (Berry et al., 2019). The colour intensity phenotype directly reflected the amount of capsanthin and its esters present. Conversely, the carotenoid content and colour retention phenotype were not related. Upon investigating carotenoid derived volatiles present in the fresh volatile data, there is clearer separation seen in relation to retention, as opposed to intensity, although intensity is still influencing the separation somewhat (Fig. S3A-B). The separation seen between the retention phenotypes gets more pronounced in dry fruit (Fig. S3D-E), therefore emphasising that colour retention is influenced more by oxidation rate than initial carotenoid concentration. Interestingly, when looking at carotenoids and apocarotenoids collectively (Fig. 3) the amount of carotenoid present at the ripe stage does not equal the amount of carotenoid derived volatiles produced. For example, R3 was found to have lower levels of β-carotene when compared to R1, and R1 and R7 had no significant difference in β-carotene levels; however, when looking at β-carotene derived volatiles R3 reflected the same β-carotene levels (higher in R1) and R7 showed a significant increase, thus again supporting the point that colour intensity phenotype and colour retention phenotype in pepper are not linked (Fig. 3).

### 3.3 Changes in the metabolome associated with colour retention

It is clear that lines with altered colour retention properties have different “volatomes”. In order to associate potential perturbations in intermediary metabolism with the “volatome” beyond quantitative and qualitative carotenoid changes, GC–MS metabolite profiling was carried out. R1, R3 and R7 represent high, low and medium retention phenotypes, respectively. Full data table can be found in Table S2.

Of the 126 compounds identified, the most abundant compounds from the non-polar extracts were the fatty acids, glycerol-1-palmitic acid and glycerol-1-stearic acid, followed by free palmitic acid and myristic acid, and unsaturated fatty acid, oleic acid. The isoprenoids, β-sitosterol, campesterol, stigmasterol, α-tocopherol, and β-amyrin were also present at high levels. The most abundant compounds in the polar extract were the sugars; fructose, glucose, inositol, sucrose, and the organic acids; citric acid, malic acid and ascorbic acid.

**Fig. 4:**
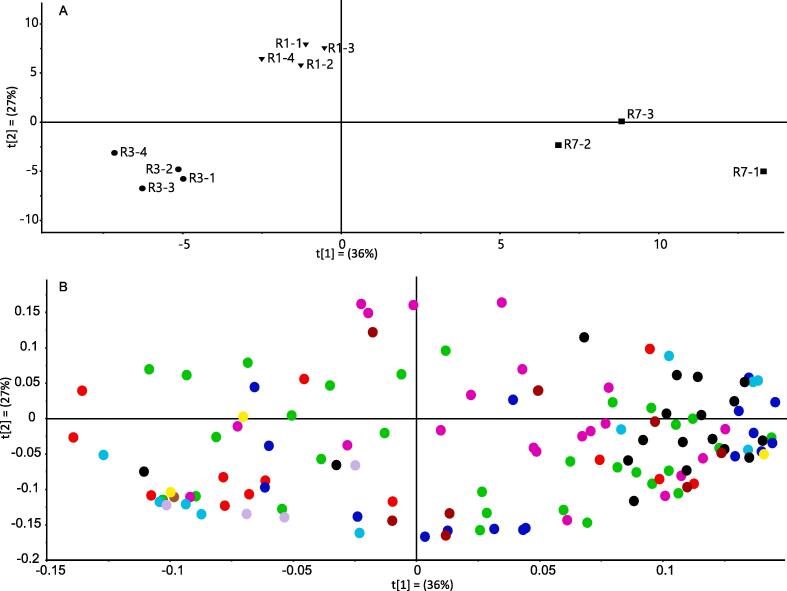
**PCA of polar and non-polar compounds in ripe fruit in selected lines.**The metabolites present in the ripe chilli pepper fruit were profiled using GC–MS. This was carried out on three selected lines with varying colour retention phenotypes. (A) PCA scores and (B) PCA loadings. The predictability of the model gave a Q2 value of 0.3 validating the approach. The key for compound classes is as follows: Amino acids (black), hydrocarbons (red), fatty acids (royal blue), unsaturated fatty acids (lilac), organic acids (purple), sugars (green), phytosterols and triterpenoids (light blue), alcohols (yellow), flavonoid (brown) other (top to bottom: dihydrocapsaicin, 4-hydroxybenzoic acid, dihydrouracil, phosphate, ethanolamine, hydroxylamine, 2-[3-Phenyl-4(3H)-quinazolinone-2-ylmethylthio]-4(3H)-quinazolinone and 2,4,6-tri-tert.-butylbenzenethiol) (dark red). The compounds present can be found in Table S2. (For interpretation of the references to colour in this figure legend, the reader is referred to the web version of this article.)

PCA revealed variation in the chemical composition of all three lines selected (Fig. 4A). The loadings indicated that unsaturated fatty acids, hydrocarbons and sterols underlined separation in the low retention line, R3. The metabolites driving separation of R1 and R7 were organic acids, amino acids and very long chain fatty acids (VLCFAs), respectively (Fig. 4B).

A heat map constructed to facilitate visualisation of the metabolite profiling data (Fig. S4) indicated that R3 was found to be higher in the majority of fatty acids when compared to R1, including C16:0-C18:0 and C18:1–2 fatty acids. C18:3 and VLCFAs were higher in R7 than R1. This shows that although the specific fatty acids measured were different, R1 was lower in fatty acids than the R3 and R7, particularly the unsaturated fatty acids. Also, linked to lipid metabolism, was the higher levels of glycerol esters found in both R3 and R7 compared to R1.

There were no obvious relationships between colour retention phenotypes and other branches of metabolism. 5 organic acids out of the 14 identified were found to have significantly higher levels in R1 (the high retention line) when compared to the other lines, but no common organic acid biosynthesis pathway could be attributed to these changes. There were 7 amino acids with lower levels in R3, compared to R1 common to alanine, cysteine and aspartic acid biosynthesis but this was unique to the low retention line (Fig. S4). Additionally, R3 had higher levels of hydrocarbons when compared to R1 but, again, this was unique to the low retention phenotype. Finally, there were also significant differences observed in the sugars, triterpenoids and phytosterols but none of these showed a correlation to colour retention phenotype (Fig. 3 and S4).

The results described above have been visualised in an alternative manner by constructing a pathway network as a method of linking the metabolite changes to specific areas of metabolism; thus, allowing the upstream and downstream effects of these changes to be taken into consideration. Both R3 and R7 (low and medium retention) were displayed on pathways constructed as a comparison to R1 (high retention) (Fig. 3). It is important to note here that the differences between selected lines of volatiles produced in fresh fruit are not linked to colour retention phenotypes, this correlation emerges once the fruit have dried and oxidation has begun.

R3 accumulated more unsaturated fatty acids in ripe fruit, such as oleic acid (C18:1) and linoleic acid (C18:2) and the corresponding glycerol monolinoleate when compared to both lines (R1 and R7). R7 accumulated more linolenic acid when compared to R1. This could suggest a more fluid membrane present in these lower retention lines, when compared to the high retention, as it is known that an abundance of unsaturated fatty acids in the cell membrane functions to keep the membrane at a more fluid state (Marangoni et al., 1996). This finding was of particular interest as it has been proposed that linoleic acid and linolenic acid are particularly prone to oxidation whereby free radicals attack the double bond (Marangoni et al., 1996, Wang and Baker, 1979). Therefore, perhaps these low and medium retention phenotypes were caused by high levels of the linoleic acid and linolenic acid present in the membranes resulting in increased lipid peroxidation which creates increased levels of free radicals. The carotenoids will be mobilised for their antioxidant properties resulting in less being stored in the fibrils and plastoglobuli; thus, contributing to a low intensity phenotype and perhaps resulting in more colour loss during storage. The fact that the volatiles derived from these two fatty acids which were found to be more abundant in the low and medium intensity lines supports this hypothesis further. Interestingly, the contrary has been shown in relation to chilling injury in post-harvest storage whereby a decrease in unsaturated fatty acids reduced the membranes flexibility in cold temperatures causing pitting and discolouration (Ge et al., 2019).

R3 was lower in VLCFAs when compared to R1 including lignoceric acid (C24:0) and cerotic acid (C26:0). These fatty acids are not very well characterised in the literature but lignoceric acid has been identified in pepper previously (Bauer et al., 2005, Conforti et al., 2007). These compounds are VLCFAs which comprise fatty acids with acyl chains over 20. They have been characterised as cuticular and epicuticular lipids which associate with triglycerides and sphingolipids (Yeats et al., 2012). Additionally, nonacosane was found to be lower in the low retention line R3; this hydrocarbon was identified as one of the key components present in bell pepper surface wax (Bauer et al., 2005). These data imply that the higher levels of VLCFA present in the higher retention lines may cause these lines to have waxier cuticles; thus protecting the carotenoids by decreasing the extent of lipid oxidation. However, more in depth analysis would need to be carried out to support this.

## 4 Conclusions

The present study aimed to develop a more rapid and logistically convenient methodology of assessing colour retention in chilli pepper. The data collectively showed that image analysis can be applied to the evaluation of colour changes during the post-harvest storage of chilli material. The use of ground material instead of whole fruit is representative and greatly reduces the storage period required for assessment. However, there are limitations, with closely related phenotypes not easily distinguishable and the approach does not take into account the role of the cuticle.

**Fig. 5:**
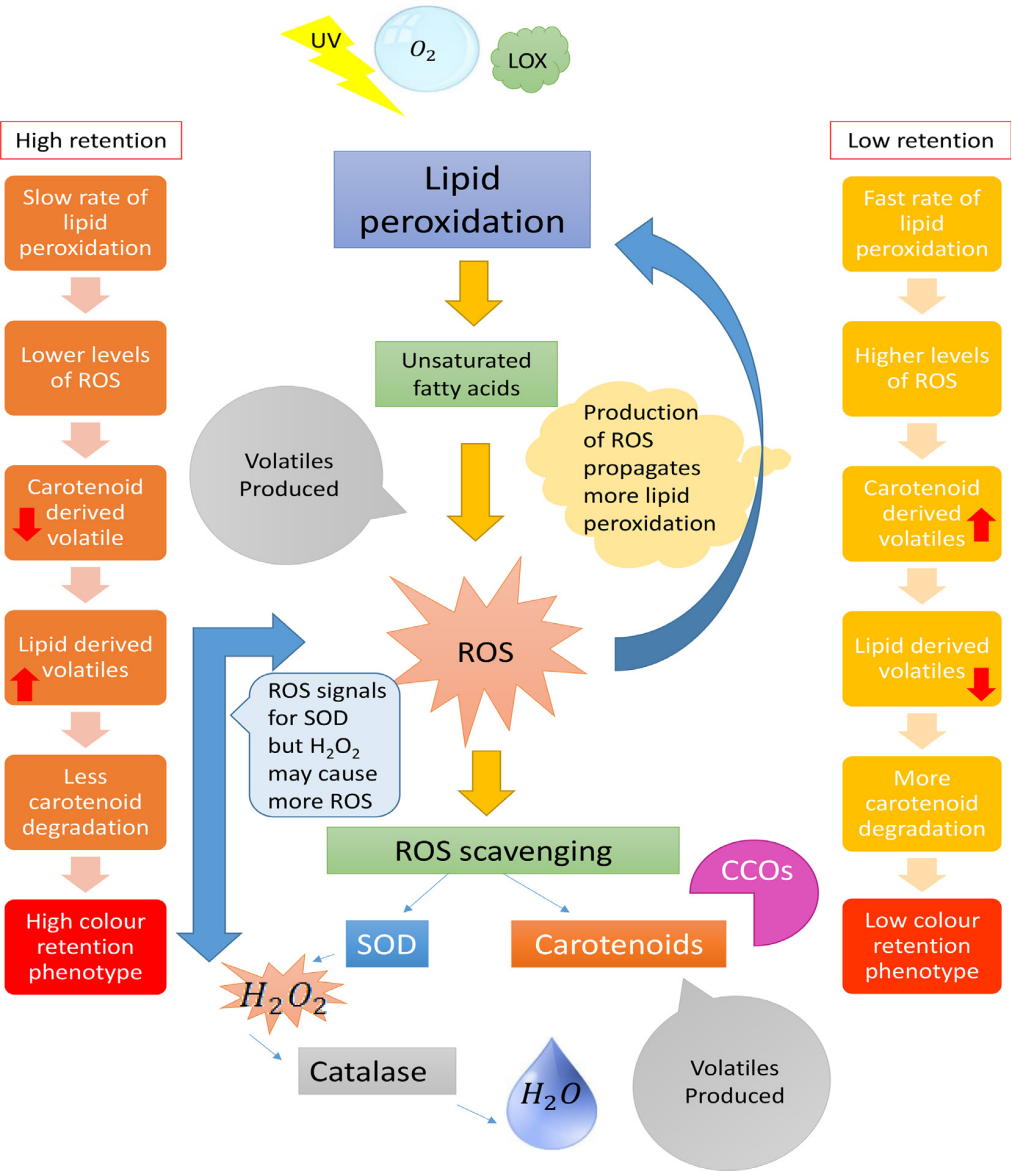
**Schematic illustration of the relationship between lipid peroxidation and carotenoid degradation present in high and low colour retention lines.** Exposure to light, oxygen and lipoxygenases (LOX) can lead to lipid peroxidation in plants. Substrates are usually unsaturated fatty acids which give rise to volatiles and reactive oxygen species (ROS) which directly effects the rate at which carotenoids present are degraded. Carotenoids can also be degraded by carotenoid cleavage oxygenase (CCOs).

Based on the hypothesis that colour loss during storage is linked to the degradation (enzymatic and non-enzymatic) of carotenoids, the volatile analysis performed indicated a role for initial lipid peroxidation dissipated by co-oxidation of carotenoids. While metabolite profiling data generated corroborated the involvement of specific fatty acids in colour retention; with higher levels of unsaturated fatty acids in low and medium retention lines. In addition, the comparative changes in VLCFA between low and high retention lines, suggests the waxy cuticle may also play a role by protecting the carotenoids from oxidation, an aspect that warrants further investigation. Fig. 5 sets out our present hypothesis, whereby the colour retention phenotype is defined by a number of factors, such as levels of LOX and CCOs, antioxidant concentrations and lipid composition. Lines differing in retention phenotype have different metabolic attributes, with the sum of oxidative pressures versus the sum of protective mechanisms are likely to ultimately define colour retention phenotypes.

Collectively these data provide important data for the future rational design of chilli varieties with improved commercial colour retention properties. Generically, these findings can also contribute to the carotenoid biofortification of staple crops targeting Low Medium Income Countries (LMICs) where provitamin A deficiency remains a major health issue.

## CRediT authorship contribution statement

**Harriet M. Berry:** Conceptualization, Data curation, Formal analysis, Investigation, Methodology, Project administration, Visualization, Writing - original draft, Writing - review & editing. **Florence Lai:** Formal analysis, Data curation. **Aniko Kende:** Formal analysis, Data curation, Methodology, Project administration, Resources. **Daniel V. Rickett:** Conceptualization, Funding acquisition, Project administration, Supervision, Writing - review & editing. **Charles J. Baxter:** Conceptualization, Funding acquisition, Project administration, Supervision, Writing - review & editing. **Eugenia M.A. Enfissi:** Conceptualization, Funding acquisition, Project administration, Supervision, Writing - review & editing. **Paul D. Fraser:** Conceptualization, Funding acquisition, Project administration, Resources, Supervision, Writing - review & editing.
