# Supplementary material for: Understanding colour retention in red chilli pepper fruit using a metabolite profiling approach
Source: Food Chem (Oxf). 2021 Jan 26;2:100013. doi: 10.1016/j.fochms.2021.100013 (PMC8991714; doi:10.1016/j.fochms.2021.100013)
Supplement: Supplementary data 1 [file mmc1.docx]

SUPPLEMENTAL MATERAL: FIGURES AND TABLES

FIGURES

**Fig. S1. Representation of the change in volatile compositions from fresh to dry.**

The percentage of each compound class was calculated for each line compared to the total amount of volatiles. The average percentage for each compound class over all the lines was determined and shown to display the changes in volatile composition in fresh and dry fruit. Error bars represent ±SE (n=6). A Student’s *t*-test was carried out to ascertain significant differences: (*P≤* 0.05 = *, 0.01 = **).

**Fig. S2. Heat map displaying the change in volatile compounds in dry fruit when compared to fresh fruit.**

Volatile analysis was carried out on a colour diversity panel to examine the differences in volatile composition between fresh and dry fruit. The changes in compounds displayed were compared to fresh fruit. The volatiles compounds identified were grouped based the chemical class the volatile was derived from: carotenoid, lipid, other-terpenoid or other. Results were the means for 6 biological replicates and significant differences were calculated using one-way ANOVA (*P* ≤ 0.05). Significant increases in metabolites in dry fruit are represented by green, significant decreases by red, no significant change by grey and not present by white.

**Fig. S3. PCA displaying the differences within fresh or dry fruit according to colour intensity or retention phenotype.**

The volatiles present in fresh fruit were visualised using PCA. Scores according to colour intensity (A) and retention (B) and PCA loadings (C). The same was carried out for the volatile present in dry fruit. Scores according to colour intensity (D), retention (E) and PCA loadings (F). Key: blue, low intensity or retention; green, medium intensity or retention; red, high intensity or retention.

|  | R3 | R7 |  | R3 | R7 |
| --- | --- | --- | --- | --- | --- |
| Alanine |  |  | 2-ketoglutaric acid |  |  |
| Aspartic acid |  |  | 2-Keto-L-gluconic acid |  |  |
| Cysteine |  |  | 4-OH-Cinnamic acid |  |  |
| GABA |  |  | Aconitic acid |  |  |
| Glutamine |  |  | Benzoic acid |  |  |
| Isoleucine |  |  | Erythronic acid |  |  |
| Lysine |  |  | Ferulic acid |  |  |
| Ornithine |  |  | Galactonic acid |  |  |
| Phenylalanine |  |  | Gluconic acid |  |  |
| Proline |  |  | Gluconic acid-1,5-lactone |  |  |
| Pyroglutamic acid |  |  | Glyceric acid |  |  |
| Tyrosine |  |  | Maleic acid |  |  |
| Hexacosanol |  |  | Malic acid |  |  |
| Octacosanol |  |  | Threonic acid |  |  |
| C12:0 |  |  | Phosphate |  |  |
| C14:0 |  |  | beta-D-Glc-(1,4)-D-Glc |  |  |
| C16:0 |  |  | beta-D-Glc-(1,6)-D-Glc |  |  |
| C17:0 |  |  | D-glycero-D-gulo-heptose |  |  |
| C18:0 |  |  | Disaccharide48.682min |  |  |
| C18:1 cis9 |  |  | Disaccharide50.399min |  |  |
| C18:2 trans9,12 |  |  | Disaccharide56.509min |  |  |
| C18:3 cis9,12,15 |  |  | Gentiobiose |  |  |
| C24:0 |  |  | Glucose |  |  |
| C26:0 |  |  | Glycerol |  |  |
| C30:0 |  |  | Glycerol-2-phosphate |  |  |
| Glycero-1-C14:0 |  |  | Glycerol-3-phosphate |  |  |
| Glycero-1-C16:0 |  |  | Inositol |  |  |
| Glycero-1-C18:0 |  |  | Inositol monophosphate |  |  |
| Glycero-1-C18:2 cis9,12 |  |  | Sedoheptulose |  |  |
| Glycero-2-C16:0 |  |  | Sorbitol |  |  |
| Glycero-2-C18:0 |  |  | Sucrose |  |  |
| Kaempferol |  |  | a-Tocopherol |  |  |
| Docosane |  |  | d-Tocopherol |  |  |
| Dodecane |  |  | g-Tocopherol |  |  |
| Hentriacontane |  |  | Phytol |  |  |
| Heptacosane |  |  | Squalene |  |  |
| Heptadecane |  |  | beta-Amyrin |  |  |
| Hexacosane |  |  | beta-Sitosterol |  |  |
| Nonacosane |  |  | Campesterol |  |  |
| Octacosane |  |  | Stigmasterol |  |  |
| Octadecane |  |  | 2,4,6-Tri-tert.-butylbenzenethiol |  |  |
| Pentadecane |  |  | Ethanolamine |  |  |
| Triacontane |  |  | Hydroxylamine |  |  |
| Tricosane |  |  | 2-[3-Phenyl-4(3H)-quinazolinone- |  |  |
| Tridecane |  |  | 2-ylmethylthio]-4(3H)-quinazolinone |  |  |

**Fig. S4. Heat map of polar and non-polar compounds in ripe fruit in selected lines.**

The metabolites in ripe fruit were profiled using GC-MS. This was carried out on selected lines varying in colour retention phenotype. The fold change and significance were calculated in comparison to the high retention line, R1. Metabolites are organised according to chemical class: Amino acids, alkane hydrocarbons, fatty acids, phosphate, triterpenoids and phytosterols and sugar like compounds. Changes are indicated by: white, not present; grey, not significant; green, significant increase; and red, significant decrease. Significance based on *P ≤* 0.05 (n=4). Compounds are organised into classes: amino acid, fatty acid, flavonoid, hydrocarbons, organic acid, phosphate, sugar, triterpenoid and phytosterol, and other.

TABLES

**Table S1. Colour intensity and retention phenotypes of the discovery panel used in this study.**

Colour phenotypes allocated by Syngenta.

|  | Image of fruit | Description of fruit | Colour intensity phenotype | Colour retention phenotype |
| --- | --- | --- | --- | --- |
| R1 |  | Small; thick walled; smooth | High | High |
| R2 |  | Long; thin walled; wrinkled skin | High | Low |
| R3 |  | Small; thin walled; smooth skin | Low | Low |
| R4 |  | Long; thick walled; smooth skin | Medium | Low |
| R5 |  | Medium; thick walled; smooth skin | Medium | Medium |
| R6 |  | Medium; thick walled; smooth skin | Medium | Medium |
| R7 |  | Long; medium walled; wrinkly skin | High | Medium |
| R8 |  | Small; thin walled; smooth skin | Medium | High |
| R9 |  | Long; thin walled; smooth skin | Medium | Low |
| R10 |  | Medium; thin walled; smooth skin | Medium | Medium |
| R11 |  | Long; thick walled; smooth skin | Medium | High |
| R12 |  | Small; thin walled; smooth skin | Medium | Low |

**Table S2. Metabolites identified in ripe chilli pepper fruit using GC-MS.**

|  | |  |  | MEAN | | | SEM | | |
| --- | --- | --- | --- | --- | --- | --- | --- | --- | --- |
| Metabolites | RT | | RI | R1 | R3 | R7 | R1 | R3 | R7 |
| 1,6-Anhydro-beta-D-glucose | | 29.27 | 1707 | 0.0007 | 0.0010 | 0.0010 | 0.0001 | 0.0002 | 0.0002 |
| 2-[3-Phenyl-4(3H)-quinazolinone- | | 9.43 | 1023 | 0.0001 | 0.0001 | 0.0001 | 0.0000 | 0.0000 | 0.0000 |
| 2-ylmethylthio]-4(3H)-quinazolinone | | | | |  |  |  |  |  |
| 2-ketoglutaric acid | | 33.05 | 1578 | 0.0120 | 0.0129 | 0.0096 | 0.0004 | 0.0012 | 0.0053 |
| 2-Keto-L-gluconic acid | | 30.96 | 1748 | 0.0011 | 0.0015 | 0.0004 | 0.0002 | 0.0006 | 0.0001 |
| 2-O-Glycerol-d-galactopyranoside | | 44.01 | 2301 | 0.0007 | 0.0005 | 0.0007 | 0.0001 | 0.0001 | 0.0002 |
| 4-hydroxybenzoic acid | | 27.22 | 1635 | 0.0002 | 0.0002 | 0.0002 | 0.0000 | 0.0000 | 0.0001 |
| 4-OH-Cinnamic acid | | 34.51 | 1941 | 0.0003 | 0.0000 | 0.0000 | 0.0001 | 0.0000 | 0.0000 |
| Aconitic acid | | 30.71 | 1751 | 0.0000 | 0.0000 | 0.0037 | 0.0000 | 0.0000 | 0.0028 |
| Alanine | | 21.23 | 1108 | 0.0001 | 0.0000 | 0.0007 | 0.0000 | 0.0000 | 0.0002 |
| Arabinose/Ribose/Xylose | | 28.07 | 1662 | 0.0015 | 0.0010 | 0.0012 | 0.0004 | 0.0003 | 0.0002 |
| Arabinose/xylose/pentose | | 31.07 | 1766 | 0.0006 | 0.0006 | 0.0004 | 0.0001 | 0.0001 | 0.0001 |
| Ascorbic acid | | 35.61 | 1938 | 0.0139 | 0.0120 | 0.0220 | 0.0070 | 0.0056 | 0.0109 |
| Aspartic acid | | 21.19 | 1425 | 0.0008 | 0.0000 | 0.0013 | 0.0001 | 0.0000 | 0.0004 |
| a-Tocopherol | | 58.83 | 3115 | 0.0592 | 0.0598 | 0.1102 | 0.0029 | 0.0033 | 0.0085 |
| Benzoic acid | | 15.47 | 1246 | 0.0010 | 0.0013 | 0.0014 | 0.0000 | 0.0000 | 0.0001 |
| beta-Amyrin | | 62.09 | 3323 | 0.0197 | 0.0253 | 0.0152 | 0.0010 | 0.0022 | 0.0009 |
| beta-D-Glc-(1,4)-D-Glc | | 47.88 | 2494 | 0.0006 | 0.0008 | 0.0011 | 0.0001 | 0.0001 | 0.0002 |
| beta-D-Glc-(1,6)-D-Glc | | 54.37 | 2534 | 0.0003 | 0.0002 | 0.0001 | 0.0001 | 0.0001 | 0.0000 |
| beta-Sitosterol | | 61.81 | 3305 | 0.0423 | 0.0725 | 0.0597 | 0.0022 | 0.0063 | 0.0043 |
| C12:0 | | 28 | 1521 | 0.0067 | 0.0046 | 0.0168 | 0.0009 | 0.0003 | 0.0015 |
| C14:0 | | 33.27 | 1719 | 0.0230 | 0.0240 | 0.0610 | 0.0023 | 0.0009 | 0.0054 |
| C15:0 | | 35.28 | 1818 | 0.0039 | 0.0030 | 0.0004 | 0.0026 | 0.0008 | 0.0000 |
| C16:0 | | 37.69 | 2041 | 0.0678 | 0.0939 | 0.0827 | 0.0084 | 0.0021 | 0.0043 |
| C17:0 | | 39.95 | 2018 | 0.0007 | 0.0009 | 0.0006 | 0.0002 | 0.0002 | 0.0000 |
| C18:0 | | 39.99 | 2238 | 0.0014 | 0.0022 | 0.0020 | 0.0001 | 0.0001 | 0.0001 |
| C18:1 cis9 | | 41.94 | 2210 | 0.0118 | 0.0167 | 0.0134 | 0.0008 | 0.0014 | 0.0008 |
| C18:1 trans9 | | 41.95 | 2090 | 0.0000 | 0.0001 | 0.0001 | 0.0000 | 0.0000 | 0.0000 |
| C18:2 trans9,12 | | 39.2 | 2082 | 0.0012 | 0.0024 | 0.0015 | 0.0002 | 0.0002 | 0.0002 |
| C18:3 cis9,12,15 | | 41.94 | 2065 | 0.0000 | 0.0000 | 0.0207 | 0.0000 | 0.0000 | 0.0011 |
| C20:0 | | 46.66 | 2322 | 0.0112 | 0.0079 | 0.0120 | 0.0042 | 0.0004 | 0.0021 |
| C22:0 | | 50.48 | 2524 | 0.0071 | 0.0107 | 0.0078 | 0.0020 | 0.0006 | 0.0008 |
| C24:0 | | 53.72 | 2725 | 0.0013 | 0.0000 | 0.0047 | 0.0001 | 0.0000 | 0.0008 |
| C26:0 | | 57.36 | 3013 | 0.0005 | 0.0000 | 0.0041 | 0.0001 | 0.0000 | 0.0007 |
| C30:0 | | 63.44 |  | 0.0008 | 0.0000 | 0.0019 | 0.0001 | 0.0000 | 0.0003 |
| Campesterol | | 60.49 | 3218 | 0.0170 | 0.0000 | 0.0315 | 0.0010 | 0.0000 | 0.0021 |
| Citraconic acid | | 18.77 | 1348 | 0.0018 | 0.0015 | 0.0015 | 0.0001 | 0.0004 | 0.0007 |
| Citric acid | | 32.46 | 1822 | 0.0995 | 0.0964 | 0.0848 | 0.0151 | 0.0113 | 0.0521 |
| Cysteine | | 25.11 | 1553 | 0.0007 | 0.0002 | 0.0006 | 0.0002 | 0.0000 | 0.0002 |
| D-glycero-D-gulo-heptose | | 44.78 | 2351 | 0.0000 | 0.0003 | 0.0003 | 0.0000 | 0.0000 | 0.0001 |
| Dihydrocapsaicin | | 50.67 | 2655 | 0.0059 | 0.0000 | 0.0000 | 0.0028 | 0.0000 | 0.0000 |
| Dihydrouracil | | 23.54 | 1498 | 0.0014 | 0.0008 | 0.0071 | 0.0006 | 0.0004 | 0.0048 |
| Disaccharide48.682min | | 48.62 | 2547 | 0.0005 | 0.0023 | 0.0005 | 0.0001 | 0.0005 | 0.0000 |
| Disaccharide50.399min | | 50.27 | 2638 | 0.0999 | 0.0801 | 0.0525 | 0.0124 | 0.0192 | 0.0145 |
| Disaccharide56.509min | | 56.31 | 2987 | 0.0002 | 0.0000 | 0.0009 | 0.0000 | 0.0000 | 0.0003 |
| Docosane | | 26.42 | 2191 | 0.0002 | 0.0003 | 0.0002 | 0.0000 | 0.0000 | 0.0001 |
| Dodecane | | 10.99 | 1194 | 0.0002 | 0.0002 | 0.0003 | 0.0000 | 0.0000 | 0.0001 |
| d-Tocopherol | | 54.97 | 9981 | 0.0007 | 0.0001 | 0.0009 | 0.0001 | 0.0000 | 0.0002 |
| Erythronic acid | | 24.8 | 1571 | 0.0003 | 0.0001 | 0.0001 | 0.0000 | 0.0000 | 0.0000 |
| Erythrose | | 35.32 | 1919 | 0.0014 | 0.0015 | 0.0009 | 0.0003 | 0.0002 | 0.0001 |
| Ethanolamine | | 16 | 1265 | 0.0003 | 0.0007 | 0.0015 | 0.0000 | 0.0001 | 0.0005 |
| Ferulic acid | | 39.12 |  | 0.0001 | 0.0000 | 0.0000 | 0.0000 | 0.0000 | 0.0000 |
| Fructose | | 34.02 | 1884 | 0.1060 | 0.1177 | 0.0993 | 0.0189 | 0.0245 | 0.0123 |
| GABA | | 24.33 | 1303 | 0.0027 | 0.0012 | 0.0032 | 0.0005 | 0.0002 | 0.0007 |
| Galactonic acid | | 36.7 | 1981 | 0.0013 | 0.0000 | 0.0005 | 0.0001 | 0.0000 | 0.0002 |
| Gentiobiose | | 53.7 | 2818 | 0.0000 | 0.0001 | 0.0002 | 0.0000 | 0.0000 | 0.0001 |
| Gluconic acid | | 36.64 | 1654 | 0.0008 | 0.0044 | 0.0008 | 0.0001 | 0.0007 | 0.0003 |
| Gluconic acid-1,5-lactone | | 34.08 | 1876 | 0.0008 | 0.0011 | 0.0021 | 0.0002 | 0.0002 | 0.0002 |
| Glucopyranose | | 34.29 |  | 0.0401 | 0.0320 | 0.0211 | 0.0073 | 0.0214 | 0.0131 |
| Glucose | | 34.35 | 1900 | 0.0670 | 0.0543 | 0.0014 | 0.0113 | 0.0108 | #REF! |
| Glutamine | | 22.66 | 1470 | 0.0003 | 0.0000 | 0.0017 | 0.0001 | 0.0000 | 0.0011 |
| Glyceric acid | | 18.18 | 1331 | 0.0002 | 0.0004 | 0.0005 | 0.0000 | 0.0000 | 0.0002 |
| Glycero-1-C14:0 | | 45.72 | 2387 | 0.0042 | 0.0062 | 0.0066 | 0.0003 | 0.0003 | 0.0002 |
| Glycero-1-C16:0 | | 49.57 | 2583 | 0.1584 | 0.2051 | 0.2157 | 0.0077 | 0.0055 | 0.0088 |
| Glycero-1-C18:0 | | 53.1 | 2768 | 0.1570 | 0.2020 | 0.1994 | 0.0074 | 0.0051 | 0.0036 |
| Glycero-1-C18:2 cis9,12 | | 52.48 | 2740 | 0.0000 | 0.0107 | 0.0000 | 0.0000 | 0.0009 | 0.0000 |
| Glycero-2-C16:0 | | 48.88 | 2546 | 0.0076 | 0.0112 | 0.0116 | 0.0008 | 0.0004 | 0.0005 |
| Glycero-2-C18:0 | | 52.43 | 2775 | 0.0070 | 0.0000 | 0.0125 | 0.0005 | 0.0000 | 0.0003 |
| Glycerol | | 16.36 | 1278 | 0.0064 | 0.0081 | 0.0088 | 0.0005 | 0.0003 | 0.0015 |
| Glycerol-2-phosphate | | 29.99 | 1724 | 0.0000 | 0.0006 | 0.0010 | 0.0000 | 0.0001 | 0.0001 |
| Glycerol-3-phosphate | | 30.98 | 1762 | 0.0072 | 0.0065 | 0.0130 | 0.0011 | 0.0014 | 0.0013 |
| Glycine | | 17.29 | 1305 | 0.0002 | 0.0002 | 0.0004 | 0.0000 | 0.0000 | 0.0001 |
| g-Tocopherol | | 56.38 | 2975 | 0.0063 | 0.0226 | 0.0071 | 0.0009 | 0.0036 | 0.0003 |
| Hentriacontane | | 58.44 | 3087 | 0.0020 | 0.0022 | 0.0030 | 0.0005 | 0.0001 | 0.0001 |
| Heptacosane | | 51.63 | 2589 | 0.0021 | 0.0116 | 0.0027 | 0.0003 | 0.0041 | 0.0004 |
| Heptadecane | | 29.34 | 1692 | 0.0002 | 0.0004 | 0.0000 | 0.0000 | 0.0000 | 0.0000 |
| Hexacosane | | 49.79 | 2589 | 0.0012 | 0.0016 | 0.0013 | 0.0001 | 0.0000 | 0.0001 |
| Hexacosanol | | 55.86 |  | 0.0000 | 0.0020 | 0.0000 | 0.0000 | 0.0004 | 0.0000 |
| Hydroxylamine | | 11.04 | 1092 | 0.0003 | 0.0005 | 0.0004 | 0.0000 | 0.0001 | 0.0000 |
| Inositol | | 38.91 | 2086 | 0.0329 | 0.0189 | 0.1132 | 0.0038 | 0.0030 | 0.0267 |
| Inositol monophosphate | | 45.86 | 2415 | 0.0070 | 0.0105 | 0.0103 | 0.0007 | 0.0014 | 0.0014 |
| Isoleucine | | 16.93 | 1294 | 0.0003 | 0.0000 | 0.0004 | 0.0001 | 0.0000 | 0.0002 |
| Itaconic acid | | 18.6 | 1343 | 0.0132 | 0.0100 | 0.0120 | 0.0011 | 0.0027 | 0.0020 |
| Kaempferol | | 57.78 |  | 0.0000 | 0.0027 | 0.0000 | 0.0000 | 0.0004 | 0.0000 |
| Lactic acid | | 9.653 | 1903 | 0.0013 | 0.0015 | 0.0024 | 0.0003 | 0.0004 | 0.0010 |
| Leucine | | 16.24 | 1274 | 0.0002 | 0.0002 | 0.0003 | 0.0001 | 0.0000 | 0.0001 |
| Lysine | | 33.25 | 1912 | 0.0000 | 0.0000 | 0.0011 | 0.0000 | 0.0000 | 0.0005 |
| Maleic acid | | 17.32 | 1302 | 0.0007 | 0.0006 | 0.0017 | 0.0000 | 0.0001 | 0.0004 |
| Malic acid | | 23.21 | 1492 | 0.0209 | 0.0159 | 0.0277 | 0.0013 | 0.0014 | 0.0180 |
| Mesaconic acid | | 20.5 | 1401 | 0.0008 | 0.0006 | 0.0010 | 0.0001 | 0.0002 | 0.0003 |
| Methionine | | 24 | 1515 | 0.0001 | 0.0001 | 0.0001 | 0.0000 | 0.0000 | 0.0000 |
| Nonacosane | | 55.14 | 2888 | 0.0021 | 0.0000 | 0.0022 | 0.0006 | 0.0000 | 0.0001 |
| Nonanoic acid | | 18.63 | 3783 | 0.0003 | 0.0003 | 0.0004 | 0.0001 | 0.0000 | 0.0000 |
| Octacosane | | 37.58 | 2788 | 0.0016 | 0.0026 | 0.0016 | 0.0002 | 0.0001 | 0.0005 |
| Octacosanol | | 51.91 |  | 0.0000 | 0.0000 | 0.0001 | 0.0000 | 0.0000 | 0.0000 |
| Octadecane | | 25.13 | 1792 | 0.0013 | 0.0020 | 0.0032 | 0.0001 | 0.0000 | 0.0007 |
| Octadecanol | | 40.64 |  | 0.0005 | 0.0006 | 0.0004 | 0.0001 | 0.0001 | 0.0000 |
| Ornithine | | 32.34 | 1747 | 0.0000 | 0.0000 | 0.0044 | 0.0000 | 0.0000 | 0.0021 |
| Oxalic acid | | 11.87 | 2365 | 0.0002 | 0.0003 | 0.0003 | 0.0000 | 0.0000 | 0.0001 |
| Pentadecane | | 17.82 | 1493 | 0.0019 | 0.0025 | 0.0025 | 0.0003 | 0.0001 | 0.0003 |
| Phenylalanine | | 27.08 | 1542 | 0.0003 | 0.0005 | 0.0008 | 0.0001 | 0.0001 | 0.0002 |
| Phosphate | | 16.28 | 1276 | 0.0503 | 0.0517 | 0.0744 | 0.0022 | 0.0064 | 0.0024 |
| Phytol | | 40.98 | 2164 | 0.0004 | 0.0000 | 0.0008 | 0.0000 | 0.0000 | 0.0001 |
| Proline | | 17.06 | 1295 | 0.0078 | 0.0227 | 0.0013 | 0.0032 | 0.0036 | 0.0002 |
| Pyroglutamic acid | | 24.05 | 1516 | 0.0034 | 0.0011 | 0.0059 | 0.0010 | 0.0003 | 0.0017 |
| Sedoheptulose | | 39.39 | 2115 | 0.0008 | 0.0013 | 0.0030 | 0.0003 | 0.0002 | 0.0011 |
| Serine | | 15.84 | 1260 | 0.0012 | 0.0015 | 0.0021 | 0.0003 | 0.0004 | 0.0007 |
| Sorbitol | | 35.14 | 1919 | 0.0004 | 0.0010 | 0.0005 | 0.0000 | 0.0001 | 0.0001 |
| Squalene | | 53.58 | 9794 | 0.0000 | 0.0011 | 0.0000 | 0.0000 | 0.0001 | 0.0000 |
| Stigmasterol | | 60.86 | 3245 | 0.0048 | 0.0242 | 0.0076 | 0.0009 | 0.0019 | 0.0003 |
| Succinic acid | | 17.7 | 1316 | 0.0002 | 0.0002 | 0.0002 | 0.0000 | 0.0000 | 0.0001 |
| Sucrose | | 50.3 | 2625 | 0.0000 | 0.0640 | 0.0000 | 0.0000 | 0.0061 | 0.0000 |
| Threonic acid | | 24.81 | 1553 | 0.0000 | 0.0006 | 0.0013 | 0.0000 | 0.0001 | 0.0006 |
| Threonine | | 19.94 | 1388 | 0.0014 | 0.0015 | 0.0034 | 0.0004 | 0.0002 | 0.0012 |
| Trehalose | | 52.02 | 2723 | 0.0001 | 0.0001 | 0.0001 | 0.0000 | 0.0000 | 0.0000 |
| Triacontane | | 56.81 | 2987 | 0.0009 | 0.0018 | 0.0008 | 0.0002 | 0.0001 | 0.0001 |
| Tricosane | | 43.8 | 2290 | 0.0006 | 0.0007 | 0.0000 | 0.0001 | 0.0001 | 0.0000 |
| Tridecane | | 16.87 | 1294 | 0.0000 | 0.0003 | 0.0000 | 0.0000 | 0.0000 | 0.0000 |
| Tryptophan | | 41.42 | 2183 | 0.0003 | 0.0005 | 0.0012 | 0.0001 | 0.0002 | 0.0006 |
| Turanose | | 35.87 | 1952 | 0.0009 | 0.0013 | 0.0007 | 0.0003 | 0.0005 | 0.0004 |
| Tyrosine | | 35.42 | 1879 | 0.0000 | 0.0000 | 0.0004 | 0.0000 | 0.0000 | 0.0001 |
| Undecane | | 10.84 | 1094 | 0.0010 | 0.0009 | 0.0007 | 0.0001 | 0.0002 | 0.0000 |
| Valine | | 14.4 | 1218 | 0.0012 | 0.0007 | 0.0019 | 0.0003 | 0.0002 | 0.0007 |
